# Design and Characterization of 3D Printed Auxetic PLA-HA Composite Scaffolds for Biomedical Application

**DOI:** 10.3390/ma19101972

**Published:** 2026-05-10

**Authors:** Mohammed Amine Benziada, Antonio Javier Sanchez-Herencia, Ismail Daoud, Hossein Besharatloo, Begoña Ferrari, Djamel Miroud, Ana Ferrandez-Montero

**Affiliations:** 1 Laboratory of Materials Sciences and Engineering (LSGM), University of Sciences and Technology Houari Boumediene, Bab Ezzouar, Algiers 16111, Algeria; ismail.daoud@usthb.edu.dz (I.D.); dmiroudlsgm18@gmail.com (D.M.); 2 Instituto de Ceramica y Vidrio (ICV-CSIC), Calle Kelsen 5, 28049 Madrid, Spain; bferrari@icv.csic.es (B.F.); aferrandez@icv.csic.es (A.F.-M.); 3 Centre d’Integritat Estructural, Fiabilitat i Micromecànica dels Materials (CIEFMA-UPC), Department of Materials Science and Engineering, Universitat Politècnica de Catalunya—BarcelonaTech, Campus Diagonal Besòs-EEBE, 08019 Barcelona, Spain; hossein.besharatloo@upc.edu; 4 Barcelona Research Center in Multiscale Science and Engineering, Universitat Politècnica de Catalunya—BarcelonaTech, Campus Diagonal Besòs, 08019 Barcelona, Spain

**Keywords:** 3D printing, biomaterials, biodegradable, mechanical properties, structure–property relations

## Abstract

**Highlights:**

- Three-Dimensional printed auxetic structures of a biocompatible PLA-40% HA composite.
- Presence of HA in the thermoplastic improves the wettability, Young modulus, and wear resistance.
- Soaking in PBS degrades the polymer, causing the composite to present worse mechanical resistance.
- Re-entrant auxetic structure demonstrated notable mechanical retention after biodegradation.

**Abstract:**

Additive manufacturing (AM) techniques are becoming key factors for repairing and replacing damaged bone. These techniques enable the customization of implants, which can be tailored to the specific area to be treated or healed. Additionally, the combination of absorbable and osteoconductive biomaterials with 3D printing could eliminate second surgeries to remove implants, which is particularly relevant in pediatric and geriatric patients. The capabilities of AM in this context affect not only the external shape but also the internal microarchitecture, where the arrangement of struts to develop complex infills enhances relevant properties such as specific strength, degradation rate, and vascularization. In this study, auxetic scaffold structures made of both polylactic acid (PLA) and a PLA-hydroxyapatite (PLA-HA) composite with 40 wt% of hydroxyapatite (HA) are designed and produced using Fused Filament Fabrication (FFF). Samples of PLA and PLA-HA were 3D printed in dense samples and with auxetic infills. In dense samples, the characterization is performed by X-ray diffraction (XRD), Raman spectroscopy, wettability tests, nanoindentation, and tribological assessments. Two auxetic cellular models have been tested after degradation in PBS media, and their microstructural, structural, and mechanical properties are analyzed. Results show that the addition of hydroxyapatite (HA) significantly improves the hydrophilicity of the PLA matrix, as evidenced by a decrease in water contact angle from 73.4 ± 4.4° to 52.6 ± 2.8° (≈28% reduction), while also enhancing its mechanical and tribological properties, with hardness increasing from 207 ± 30 MPa to 241 ± 28 MPa (≈15%) and Young’s modulus from 4.08 ± 0.55 GPa to 6.24 ± 0.61 GPa (≈53%). Additionally, biodegradation of PLA-HA composites reveals a significant reduction in mechanical properties after 15 days, while the auxetic re-entrant structures mostly retain their shape during compression testing.

## 1. Introduction

Biomedical research is increasingly focusing on the development of advanced, patient-specific biomaterials with innovative properties. Advances in materials science—including polymers, metals, and composites—are supporting numerous innovative medical applications such as implants, prosthetics, regenerative medicine, nanotechnology, biosensors, and tissue engineering materials [1,2,3]. Bone tissue engineering requires substitute materials that meet a number of critical criteria. From a mechanical standpoint, sufficient strength is needed to bear physiological loads [4,5,6], and adequate impact absorption capacity helps dissipate energy and prevent failure under dynamic solicitations [7]. Progressive biodegradation is equally important, as it allows the scaffold to be gradually replaced by newly formed bone tissue [8]. Beyond mechanical performance, the substitute must exhibit biomimetic properties that mimic the native bone micro environment [9,10], along with high biocompatibility to ensure cell viability and proper tissue integration [11]. From a structural perspective, interconnected porosity is a key feature that facilitates cell ingrowth and vascularization [12], while architectures with enhanced flexibility, such as auxetic geometries, offer improved mechanical adaptability [13,14,15]. Lastly, surface wettability, particularly hydrophilicity, is a determining factor in promoting cell adhesion and nutrient transport [16,17].

On the other hand, auxetic structures, characterized by their ability to expand transversely when stretched, exhibit enhanced toughness, resilience, shear strength, and energy absorption, making them promising candidates for biomedical applications [18,19]. They offer several structural advantages like high porosity and flexibility [20], benefiting implant design (sutures, stents, flexible devices, dressings, and especially bone tissue engineering) and improving integration, strength, and regeneration [21,22,23,24,25,26,27]. The relevance of auxetic structures in enhancing mechanical resistance has been reported for various materials, particularly in construction and transportation applications. Ru Zhong et al. [28] considered two different auxetic structures made of aluminum and filled with concrete. Compression test of these samples shows an initial peak stress and a stable platform stress, which is more relevant if they superpose the two structures in a layered arrangement. Zeyao Chan et al. [29] reported that 3D-printed auxetic honeycomb structures made with nylon exhibit high stiffness and energy absorption capability. Kunyuan Li et al. [30] developed a re-entrant star-shaped auxetic metamaterial that was manufactured on stainless steel by SLM, showing enhanced stiffness and strength.

The strong development of additive manufacturing techniques has boosted the fabrication and characterization of parts with auxetic internal structures. Among the AM techniques, Fused Filament Fabrication (FFF) is one of the most extended, due to its low cost, accessibility, and relative ease-of-use. It is based on the thermal extrusion of a thermoplastic polymer through a heated, computer-controlled nozzle, which can form complex shapes, dense or with different infills (like porous auxetic scaffolds), with high precision [31,32]. Many thermoplastic polymers are used to print pieces by FFF, each one with different thermal, mechanical, or biological behavior. Those thermoplastics can be functionalized by the incorporation of inorganic particles to fabricate composites with specific properties. For example, the addition of graphene or nanosized TiO_2_ will provide to the printed pieces with electrical conductivity or photocatalyst behavior [33,34].

Polymers and bioceramics play an important role in biomedical research, particularly bone tissue engineering [35]. Within these categories, polylactic acid (PLA) and hydroxyapatite (HA) are especially interesting. PLA is a biodegradable polymer derived from renewable resources that exhibits good biocompatibility and is resorbable [36], making it an ideal material for a range of biomedical. Its elastic modulus is comparable to human bone, making it an attractive material for bone replacement [37]. On the other hand, HA is an inorganic compound naturally found in human bones and teeth. It is known for its high biocompatibility and its ability to stimulate bone regeneration. However, pure hydroxyapatite ceramics have weak mechanical properties, stemming from their fragility, low strength, and poor resistance to fatigue [38]. PLA and HA are often combined and printed by FFF to leverage their complementary properties in various biomedical applications [38,39,40]. In this sense, Sukumar et al. [41] developed and tested PLA and hydroxyapatite-reinforced PLA (HPLA) filaments for 3D printing using the material extrusion method. Mechanical testing on ASTM-standard tensile, flexural, impact specimens, and bone scaffolds showed that HPLA composites exhibited superior strength and crystallinity, supporting their potential for biomedical applications. Ibrahim et al. [42] printed cylindrical auxetic artificial bone scaffolds using HA-PAN and PLA composites. The re-entrant geometry significantly influenced the compressive strength and direction-dependent stiffness, showing strong potential for bone defect restoration. Wang et al. [43] designed 3D-printed PLA/nano-hydroxyapatite (n-HA) composite scaffolds using FFF technology, achieving tunable mechanical strength by adjusting n-HA content. In vitro and in vivo tests confirmed the superior biocompatibility and osteogenic potential of the scaffold made with the hybrid composite compared to pure PLA, making it a promising material for large bone defect repair. Recently, the effectiveness of the colloidal process in producing biocompatible PLA/HA feedstock with enhanced homogeneity of the bioceramic phase (HA) within the polymeric matrix has been demonstrated [44]. In a previous study conducted by the authors [45], the biological properties of composite scaffolds composed of colloidally dispersed PLA and 40 wt% HA, with two different infill densities (50% and 70%) were investigated. The results demonstrated that both scaffolds exhibit good biocompatibility, are non-toxic, and show promising osteogenic potential in vitro. Under strictly controlled experimental conditions, HA-PLA scaffolds with the two infills proved to be biocompatible and bioactive.

While significant progress has been made in the development of PLA-HA composites for bone tissue engineering, several critical aspects remain insufficiently addressed in the literature. Most studies focusing on PLA-HA composites have investigated either their material properties or their biological performance independently, without coupling them with auxetic structural designs. Moreover, although auxetic scaffolds have demonstrated promising mechanical advantages, their fabrication using biocomposite filaments such as PLA-HA via FFF, and the subsequent evaluation of their in-vitro biodegradation behavior, remain largely unexplored. In particular, the combined effect of HA reinforcement on the wettability, tribological performance, and mechanical stability of auxetic re-entrant scaffolds, as well as the evolution of these properties during degradation in a simulated physiological environment, has not been systematically investigated, to the best of the authors’ knowledge.

To address these gaps, the present work proposes a comprehensive investigation combining the physicochemical and mechanical properties of a PLA/40 wt% HA composite with two auxetic re-entrant infill structures, fabricated via Fused Filament Fabrication (FFF) 3D printing. The composite material is thoroughly characterized using XRD, Raman spectroscopy, water contact angle measurements, nanoindentation, and tribological testing. Subsequently, the two auxetic scaffold geometries are subjected to in vitro biodegradation in a simulated physiological medium and evaluated in terms of microstructural evolution, structural integrity, and mechanical behavior, thereby providing new insights into the suitability of auxetic PLA-HA scaffolds for load-bearing bone tissue engineering applications.

## 2. Materials and Methods

### 2.1. Starting Materials

Polylactic acid (PLA) and Polylactic acid-hydroxyapatite composite (PLA-HA) filaments were purchased from COLFEED4Print (1.75 Ø, Madrid, Spain). The PLA-HA filament composition consisted of PLA with 40 wt% of HA particles highly dispersed within the thermoplastic matrix. Phosphate Buffered Saline (PBS-P3813) was supplied from Sigma Aldrich with a pH of 7.4. The physical properties of the filament, provided by the fabricant, had a density of 1.63 g/cm^3^, a glass transition temperature of 62 °C, and a melting temperature of 156 °C.

### 2.2. Samples Design and Printing

**Figure 1:**
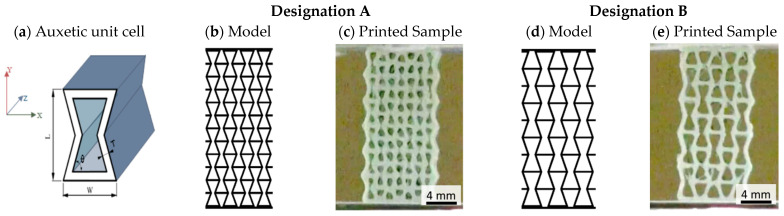
Geometric representation of the re-entrant auxetic unit cell (**a**) and sketched and printed samples of type A (**b**,**c**) and type B (**d**,**e**).

Three different types of samples were printed: two prismatic auxetic scaffolds and a dense (100% infil) cylinder. The two auxetic models being investigated featured a re-entrant cellular model. Using CAD software (FreeCAD 1.1.1), two distinct unit cell geometries were created, referred to as model A and model B. The two unit cells differ in the length (L) and width (W) of their walls in the 2D dimensions (Y and X axis, respectively), and in cells density on the XY plane. In both cases, the dimensions of the whole sample were 10 mm × 10 mm × 18 mm. Table 1 collects the geometric parameters for the scaffold geometries A and B, including the length (L), width (W), angle (θ), and wall thickness (T) of the re-entrant auxetic cell, as illustrated in Figure 1a. Dense cylinders (100% infil), referred to as model C, were also printed, with a diameter of 10 mm and a height of 2 mm for material characterization purposes before and after biodegradation test.

The printed auxetics scaffolds were carried out using a Creator Pro 2 3D printer (FLASHFORGE, Hangzhou, China) with a direct drive extruder. The printing parameters included an extruder speed of 10 mm/s, nozzle temperature of 155 °C, and bed temperature of 40 °C. Printing conditions were the same for the two geometry cells (A and B). Dense compacts underwent surface preparation with SiC paper up to 1200 grit, followed by diamond polishing to achieve a 1 µm finish.

### 2.3. XRD Analysis

Compacts were characterized by X-ray diffraction (XRD) using a PANalytical Empyrean diffractometer with Cu radiation (λ = 1.54 Å). Tests were conducted at 40 kV and 15 mA, with 2θ scanned from 10° to 70° in 0.03° steps. The crystallite size was estimated at approximately 35.77 nm using the Scherrer equation [46]:

(1) $$D=\frac{k\;\lambda }{\beta cos\theta }$$

where *D* is the crystalline size, *λ* the wavelength of the X-ray (0,154 nm), *β* is the half-width of the diffraction band (radians), *θ* is the Bragg diffraction angle (peak position in radians), *k* = 0.95 is the shaper factor. The crystallinity degree of the PLA-HA composite was estimated from the XRD pattern using the following equation [47]:

(2) $$Cry(\%)=\frac{S_{Crystalline}}{S_{total}}\times 100$$

where *S_Crystalline_* represents the cumulative area under the crystalline diffraction peaks and *S_total_* is the total area under the diffraction pattern, including both crystalline and amorphous contributions. The microstrain of the composite was determined from X-ray diffraction (XRD) patterns before degradation and after immersion PBS for 15, 30 and 60 days using the Williamson6hall method, according to the following equation:

(3) $$\beta \cos\theta =\frac{K\;\lambda }{D}+4\;\varepsilon \sin\theta$$

where β is the full width at half maximum (FWHM) of the diffraction peaks in radians, θ is the Bragg angle, *k* is the shape factor (~0.9), *λ* is the X-ray wavelength, *D* is the crystallite size and ε is the microstrain [48].

### 2.4. Density Analysis

The density of the materials PLA, HA and PLA-HA was measured using a helium pycnometer (Micromeritics, ACCUPYC 1340, Norcross, GA, USA). The obtained density values were compared to the theoretical density ($\rho_{th}$), and the porosity (*P*) of the dense printed sample was calculated using the following formula [49]:

(4) $$\rho_{th}=\rho_{PLA}.f_{PLA}+\rho_{HA}.f_{HA}$$

(5) $$P\left(\%\right)=(1-\frac{\rho_{He}}{\rho_{th}})\cdot 100\%$$

where $\rho_{PLA}$ and $\rho_{HA}$ represent the densities of PLA and HA, which are 1.24 g/cm^3^ and 3.16 g/cm^3^, respectively. *f_PLA_* and *f_HA_* are the mass fractions of PLA and HA, set at 80% and 20%, respectively. $\rho_{He}$ is the experimentally obtained density, and $\rho_{th}$ is the theoretical density.

### 2.5. Raman Spectroscopy

Raman spectroscopy was performed with a Spectrometer (RENISHAW CENTRUS 0LL824, Gloucestershire, UK), operating at 25 mW and 532 nm laser excitation, scanning from 500 to 3500 cm^−1^.

### 2.6. Wettability Test

Surface wettability was assessed on dense 3D printed samples of pure PLA and PLA-HA composites using a Ramé–Hart Goniometer/Tensiometer (model 590). A 5 µL water droplet was carefully deposited at five distinct positions on each sample surface. The reported contact angle value corresponds to the mean of the five measurements, and the error bars represent the standard deviation.

### 2.7. Nanoindentation

Nanoindentation tests on a PLA and a PLA-HA composite samples were carried out with Anton Paar STeP E600 equipment, using a Berkovitch diamond tip. The test was performed using a single measurement matrix of 4 × 4 indentations with 50 μm spacing, a penetration depth of 2000 nm, a loading/unloading rate of 15 μm/min, and a 20 s dwell time. Hardness (HIT) and Young’s modulus (EIT) were calculated using the Oliver and Pharr model following the standards of instrumented indentation technique. Each value provided corresponds to the average of the 16 measurements, and the error bars represent the standard deviation.

### 2.8. Tribological Test

Dry tribological tests were performed using a CSM-tribometer in accordance with the ASTM standard G99-17 (Method for Wear Testing with a Pin-on-Disk Apparatus) [50], using a steel ball (6 mm Ø) as the counter material. The friction coefficient (CF) was continuously recorded during the test by the tribometer computer system. After testing, samples were cleaned thoroughly, and weight loss was measured with an AND-GR200 balance (accuracy 10^−4^ g). The wear rate was then calculated using the specified equation:

(6) $$WR=\frac{m}{\left(\rho \cdot D\cdot F\right)}$$

where *WR* is the specific wear rate (mm^3^·m^−1^·N^−1^), *m* is the mass loss (g), ρ is the density (g/cm^3^), *D* is the sliding distance (m), and *F* is the normal load (N). The tests were conducted at room temperature with a sliding speed of 10 cm/s over a 200 m distance in a circular path with a 5 mm radius. Initially, the applied load varied between 5, 7, and 10 N. In the second set of tests, the load was fixed at 7 N while the sliding speed was varied from 10 to 20 cm/s in 5 cm/s increments. The surface wear topography was analyzed using a 3D profilometer (Bruker Contour GT-K, Tucson, AZ, USA) after the tribological tests to evaluate the materials’ friction and wear behavior. Three independent repetitions (n = 3) were conducted under each test condition (applied load and sliding speed). For the friction coefficient and wear rate plots, the error bars represent the standard deviation calculated from the three measurements obtained for each parameter set.

### 2.9. In Vitro Biodegradation Test

For the in vitro biodegradation tests, the auxetic models A and B were immersed in 40 mL of PBS and model C, which is a dense cylindrical material with dimensions of 5 × 2 mm in 10 mL of PBS. The initial weight to PBS volume ratio was of 0.034, 0.027, 0.023 for models A, B, and C respectively. To simulate biological conditions, the tubes were placed in a water bath maintained at 37 °C; following ASTM F1635 (Test Method for in vitro Degradation Testing of Hydrolytically Degradable Polymer Resins and Fabricated Forms for Surgical Implants) [51] PBS solutions were replaced weekly. The initial weight of each sample was recorded before testing. During immersion, samples were periodically removed to measure swelling (SW). After drying, their weight was used to assess degradation. Swelling was determined by weighing the samples immediately after removal from PBS, and degradation was assessed by their weight after drying. Swelling (SW) and mass loss (ML) were calculated using the following equations:

(7) $$Sw\left(\%\right)=\frac{M_{f}-M_{i}}{M_{i}}\times 100$$

(8) $$M_{L}\left(\%\right)=\frac{M_{i}-M_{df}}{M_{i}}\times 100$$

where M_i_ is the initial mass of the sample before immersion, M_f_ is the sample mass after removal, and M_df_ is the mass of the dried sample after immersion. Measurements were taken at 0, 15, 30, and 60 days during in vitro degradation. Results represent the average of three samples, with standard deviation included to ensure reproducibility.

Surface analysis using scanning electron microscopy (JEOL JSM-7610FPlus, Tokyo, Japan) was also conducted after in vitro biodegradation tests on samples hand polished with 1200-grit sandpaper, and later with a polishing cloth containing diamond of 1 µm.

### 2.10. Compression Test

The mechanical properties of the printed samples after in vitro biodegradation tests were evaluated using both compression and hardness tests. The compression test was conducted on auxetic scaffolds (A and B) using a universal testing machine (Microtest EM2/200/FR Madrid, Spain) with parallel plates, following the ASTM-695 standard (Method for Compressive Properties of Rigid Plastics) [52]. The materials tested were PLA and PLA-HA. The test was performed at a travel speed of 1 mm/min and a compression rate of 100% under environmental conditions of 25 °C. Additionally, Vickers hardness was measured per ASTM E384-22 (Method for Microindentation Hardness of Materials) [53] for a dense sample (model C), under a 0.1 HV load and a 10 s dwell time (Shimadzu G-17, Japan). Ten tests per sample were performed, with results averaged.

### 2.11. Statistical Analysis

All experimental results are expressed as mean values ± standard deviation (SD), in accordance with the requirements of each corresponding test standard. Statistical analysis was performed to assess the reproducibility and reliability of the obtained data across all characterization techniques, including wettability, nanoindentation, tribological testing, and mechanical evaluation after biodegradation.

## 3. Results and Discussion

### 3.1. Structural Characterization of the Dense Printed PLA and PLA-HA

#### 3.1.1. Density Analysis

Density of PLA and PLA-HA samples shows values of 1.23 ± 0.01 g/cm^3^ and 1.60 ± 0.01 g/cm^3^, respectively. The data clearly indicate that adding HA increases the material’s density. Marzuki et al. [54] prepared HA/PLA feedstock with different HA contents, and their density analysis showed a proportional increase in density with higher HA levels. Furthermore, incorporating 40 wt% HA did not significantly change the porosity rate of the dense printed samples, which ranged between 0.51% and 1.19%.

#### 3.1.2. XRD Analysis

**Figure 2:**
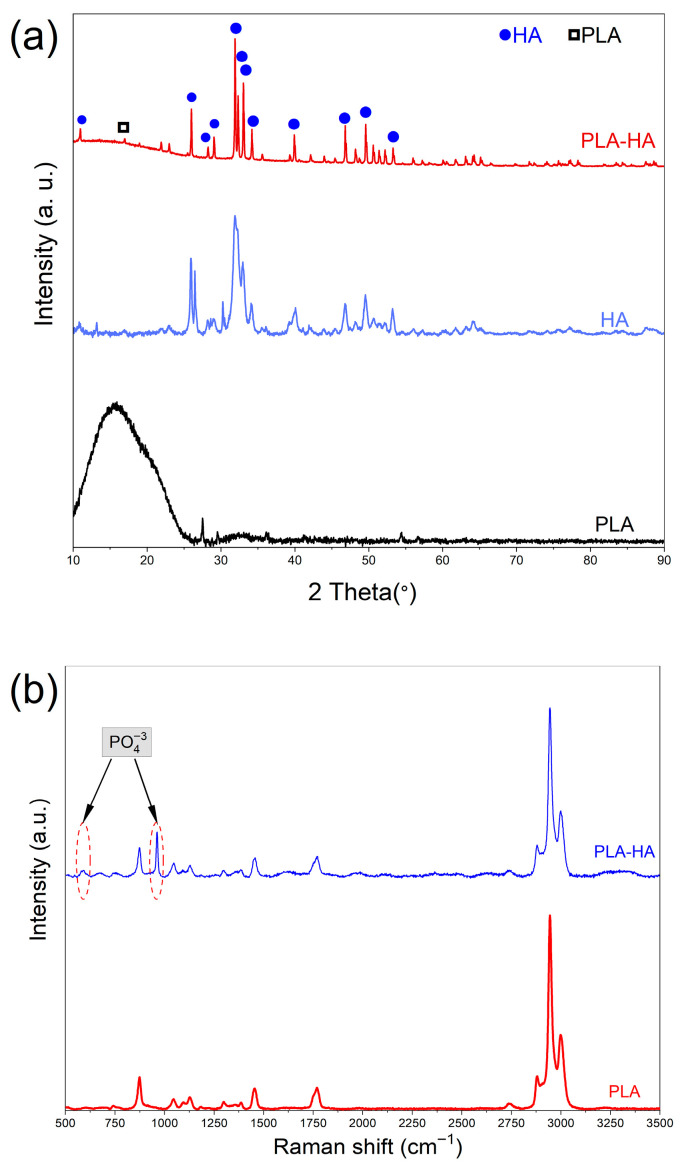
(**a**) XRD patterns of neat PLA, HA powders, and PLA-HA composite; (**b**) Raman spectra of neat PLA and PLA-HA.

Figure 2 illustrates the XRD spectra of HA powder, 3D printed dense neat PLA, and PLA-HA composite. For the neat PLA, the XRD profile shows a prominent broad diffraction peak at 2θ ranging from 10° to 26°, indicating the semicrystalline nature of PLA. Similar results have been reported for 3D printed PLA materials [55].

The HA powder spectrum shows sharp characteristic peaks at different 2θ positions 10.80, 25.87, 28.11, 28.84, 31.69, 32.16, 32.81, 34.02, 39.69, 46.60, 49.43, and 53.20, respectively, corresponding to the (100), (002), (102), (120), (121), (112), (030), (022), (310), (222), (123), and (004) diffractions planes identified by the ICDD-076-0694 as related to hexagonal symmetry. Meanwhile, the composite displays many peaks attributed to the crystalline structure of HA [40]. Additionally, a broad diffraction peak can be seen between 13° and 25°, which is related to scattering from the PLA matrix. Within this region, a diffraction peak at 18.11°, assigned to the (110) plane of the α-phase of PLA, can also be observed [56,57]. This confirms the successful incorporation of hydroxyapatite into the PLA matrix, resulting in a biphasic material composed of crystalline HA and predominantly amorphous PLA.

The degree of crystallinity of the PLA-HA composite was estimated using the peak area integration method, yielding a value of 92.88%. This high crystallinity is consistent with the dominant contribution of the highly crystalline HA phase within the amorphous PLA matrix, as evidenced by the sharp and well-resolved HA diffraction peaks observed in the pattern. This result confirms that the FFF processing did not significantly alter the crystalline structure of the hydroxyapatite phase. The quantification of crystallinity using the XRD area method is limited by several factors. The separation between crystalline peaks and the amorphous halo strongly depends on the accuracy of baseline determination and peak deconvolution, which remain partly subjective and operator-dependent. In addition, preferred grain orientation can artificially enhance specific diffraction peaks, leading to significant errors in the calculated crystallinity. Peak overlap from multiple phases further complicates phase identification and makes reliable deconvolution more difficult. Peak broadening in nanostructured or partially amorphous materials reduces the distinction between amorphous and crystalline contributions, thereby decreasing the accuracy of the method. Finally, the limited resolution of certain experimental approaches, particularly in situ measurements, makes it difficult to detect small changes in crystallinity, resulting in reduced sensitivity [58].

#### 3.1.3. RAMAN Spectroscopy

The bonding configuration of the neat PLA and PLA-HA composite was investigated using Raman spectroscopy, as shown in Figure 2b. In the PLA spectrum, several distinctive bands appear at 2997, 2944, and 2878 cm^−1^, corresponding to the stretching (both asymmetric and symmetric) vibrations of the C-H bonds in the polymer chain. Additionally, a peak at 1768 cm^−1^ can be attributed to the stretching vibration of the C=O bond. The bands at 1455 and 873 cm^−1^ are assigned to the asymmetric deformation vibration of CH_3_ and the stretching vibration of C-COO, respectively. Compared to the spectrum of the neat material, the PLA-HA composite with 40 wt% HA loading exhibits all the characteristic polymer bands. Notably, a new band at 962 cm^−1^ is observed, which can be attributed to the [PO_4_]^−3^ stretching vibration from the HA structure. This band provides strong evidence for the presence of the inorganic compound within the polymer matrix. Furthermore, an additional band at 590 cm^−1^, corresponding to the asymmetric deformation vibration of [PO_4_]^−3^ in the HA structure, is also evident [57,59]. The band at 1126 cm^−1^ is attributed to the asymmetric deformation vibration of CH_3_ groups, while the band at 1048 cm^−1^ corresponds to the C–CH_3_ stretching mode, both being characteristic of the PLA molecular backbone [60].

#### 3.1.4. Wettability Measurement

**Figure 3:**
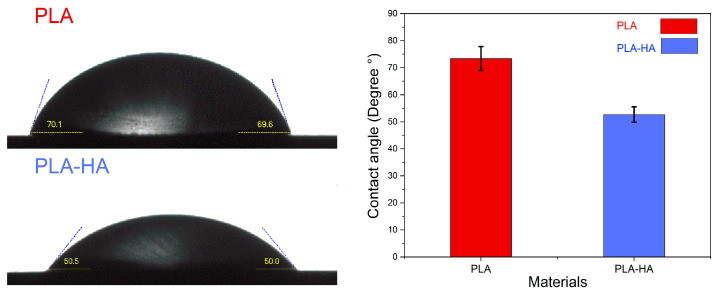
Contact angle of 3D printed dense neat PLA and PLA-HA composite.

The wettability test was conducted to determine whether a material’s surface is hydrophilic or hydrophobic and to evaluate the effect of adding 40% HA to PLA. Figure 3 displays the contact angle measurements of water droplets on both PLA and PLA-HA composites. The results indicate that both printed materials, PLA and PLA-HA, had contact angles of 73.4 ± 4.4° and 52.6 ± 2.8°, respectively. A contact angle less than 90° indicates that the surface is hydrophilic [61]. Therefore, the results confirm that both PLA and PLA-HA are hydrophilic and that the presence of hydroxyapatite (HA) particles in the PLA-HA composite caused a significant decrease in the contact angle. This increase in the hydrophilicity of the PLA material by the addition of HA particles can be explained by the presence of hydroxyl (-OH), whose polarity can form hydrogen bonds with water molecules, enhancing the material’s affinity for water, and phosphates groups (-[PO_4_]^−3^) that also contribute to polarity and can interact with water through ionic and hydrogen bonding. The combination of HA and PLA in a composite reduces their interactions caused by hydrophobic forces, leading to better dispersion of HA within the PLA matrix and greater exposure of OH groups on the composite surface [62]. Bernardo et al. [63] observed similar results, showing that the addition of HA improved the hydrophilicity of the PLA-HA composite scaffolds. It has to be noted here that improvement on the surface hydrophilicity and roughness of PLA facilitates a better protein adsorption and consequently enhances the cell adhesion and proliferation [64].

#### 3.1.5. Nanoindentation Test

**Figure 4:**
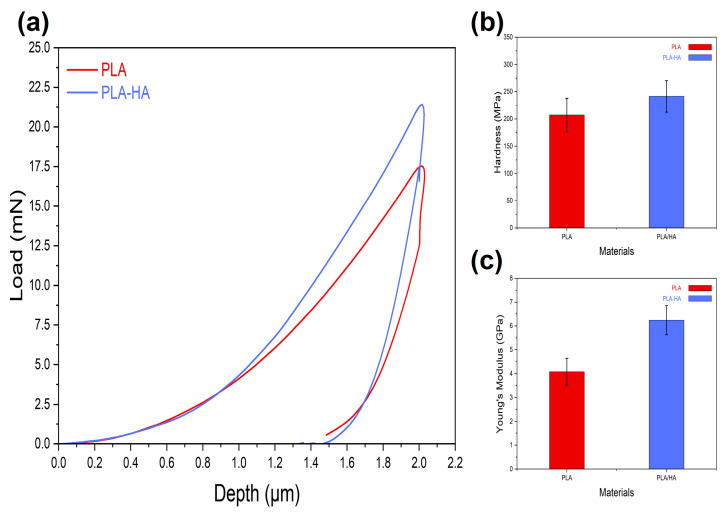
Nanoindentation results for dense 3D printed PLA and PLA-HA composite (**a**) typical comparative load-displacement curves, (**b**) Hardness, and (**c**) Young’s Modulus.

The results of the nanoindentation test provided valuable insights into the mechanical properties of the material. Key parameters measured during the test are hardness and Young’s modulus, which are critical indicators of a material’s ability to resist deformation and its overall stiffness. The results of the nanoindentation test for the dense printed neat PLA and PLA-HA composite are shown in Figure 4. The comparative load-displacement curves (Figure 4a) show that the PLA-HA composite requires a higher load to reach a specific depth compared to neat PLA, suggesting greater resistance to plastic deformation against the indenter displacement into the printed composite.

The mean values of hardness and elastic modulus from nanoindentation data are given in Figure 4b and Figure 4c, respectively. It can be seen that the hardness of neat PLA is 207 ± 30 MPa, while the PLA-HA composite exhibited a higher hardness of 241 ± 28 MPa. This 15% increase in hardness is attributed to the presence of the hard ceramic hydroxyapatite (HA) particles. Furthermore, the presence of ceramic particles impedes dislocation motion within the composite material, thereby enhancing both its hardness and stiffness [65].

Regarding Young’s modulus, the results indicate that PLA exhibited a value of 4.08 ± 0.55 GPa, whereas the PLA-HA composite achieved a higher modulus of 6.24 ± 0.61 GPa. Similar enhancements in mechanical properties were reported by Grigora et al. [66] for 3D printed PLA/montmorillonite (MMT) nanocomposite with the addition of nano HA as coating. Davila et al. [57] conducted a study on 3D printing using PLA-HA composites, where the concentration of hydroxyapatite (HA) was progressively increased up to 13 wt.%. They reported that both Young’s modulus and hardness increased as a function of the HA concentration. The standard deviation results for hardness and Young’s modulus of the PLA-HA composite are higher than those of pure PLA, due to the heterogeneous surface of the composite. In summary, these enhancements are crucial for potential biomedical applications, where mechanical integrity and performance are essential.

#### 3.1.6. Tribological Test

**Figure 5:**
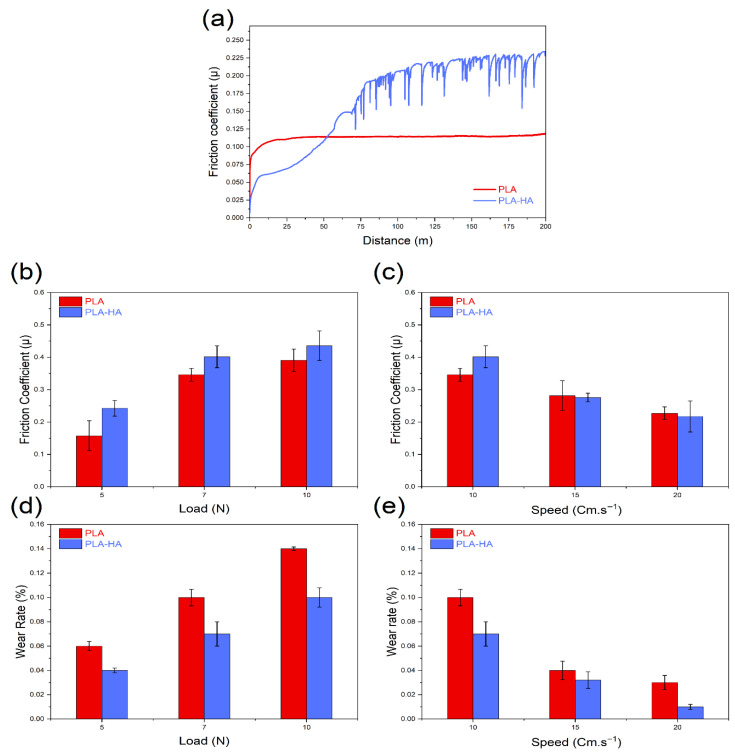
(**a**) Friction coefficient of dense printed neat PLA and PLA-HA composite under 5 N applied load and a sliding speed of 10 cm·s^−1^; (**b**,**c**) Influence of applied load and sliding speed on the coefficient of friction and (**d**,**e**) wear rate.

Wear tests were conducted to evaluate the tribological behavior of dense printed PLA and PLA-HA composites under varying experimental conditions, specifically load and speed. Figure 5 illustrates the friction coefficient (COF) curves for these dense samples under dry wear conditions (5 N load, 10 cm/s sliding speed, and 200 m sliding distance). The COF of both materials exhibits an initial increase during the test, followed by stabilization in a steady-state phase, marking the end of the run-in period.

For neat PLA, this steady-state stage is reached after approximately 25 m of sliding, whereas the PLA-HA composite requires about 100 m to stabilize. Interestingly, the steady-state COF curve for the PLA-HA composite displays notable fluctuations compared to the smooth profile observed for neat PLA. This behavior is attributed to changes in surface roughness caused by the incorporation of hard HA particles. The hard HA particles present on the composite surface act as asperities, creating obstacles that hinder the sliding of the counterpart ball. This interaction leads to increased friction and contributes to the observed fluctuations in the COF during the steady-state phase of the wear test for the PLA-HA composite. Additionally, the accumulation of wear debris can also contribute to an increase in the COF during the test [67]. Figure 5b–e depicts how the friction coefficient and wear rate change with applied load and sliding speed. A constant sliding speed of 10 cm/s increases the load results in a higher friction coefficient for both PLA and PLA-HA. Notably, the PLA-HA composite shows a greater friction coefficient than pure PLA, due to the presence of hydroxyapatite, which boosts friction. 

In the second test, the load was fixed at 7 N, while sliding speeds varied (10, 15, and 20 cm/s). Results (Figure 5c) indicate that increasing sliding speed reduces the friction coefficient for both materials. At 10 cm/s, the PLA-HA composite has a higher friction coefficient than pure PLA, but at 15 and 20 cm/s, the composite’s friction coefficient drops significantly. This suggests that the composite resists sliding more at lower speeds but produces less friction at higher speeds.

The wear rate also increased with load (Figure 5d). However, PLA’s wear rate is much higher than that of the PLA-HA composite at all loads, highlighting hydroxyapatite’s role in improving wear resistance. Additionally, higher loads generate more heat, softening the contact surfaces, which increases sliding resistance and raises both the friction coefficient and wear rate for PLA and PLA-HA. Similar behavior was observed by Rachaiah et al. [68] in 3D printed PLA-graphene composites.

At varying sliding speeds, the wear rate shows a noticeable decrease with increasing speed (Figure 5e). This trend indicates that higher speeds reduce adhesion effects at the contact interface (sample-ball), leading to less pronounced wear.

**Figure 6:**
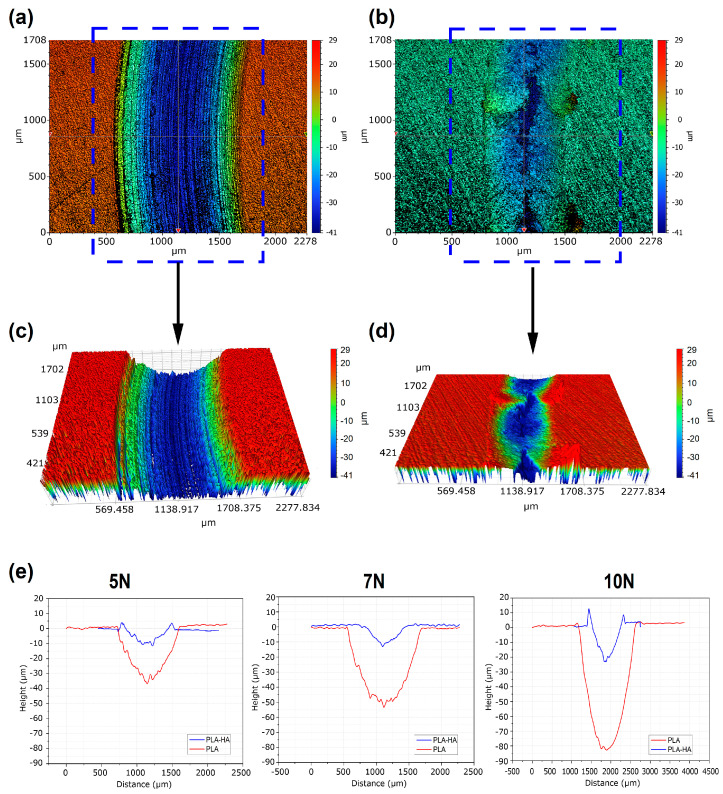
3D optical profilometer images of the worn surface after wear test under applied load of 7 N and a sliding speed of 10 cm/s for (**a**,**c**) PLA and (**b**,**d**) PLA-HA composite; (**e**) Wear path profiles of the dense 3D printed PLA and PLA-HA composites after wear test under various applied loads (5, 7, and 10 N) and at a sliding speed of 10 cm/s.

The worn surface topography was examined using a 3D profilometer to evaluate how the materials behave under friction and wear. Figure 6a–d display the worn surface profiles after a tribological test with 7 N load, 10 cm/s sliding speed, and 200 m sliding distance. PLA shows a smooth surface with sliding grooves, indicating abrasive wear. The PLA-HA composite, however, has an uneven surface with material accumulation at the edges, suggesting that the addition of HA promote a transition from abrasive to adhesive wear. HA also enhances the resistance to material removal, making the composite more resistant to flow under the pressure of the ball.

Figure 6e illustrates wear path profiles of dense 3D printed PLA and PLA-HA composites after testing under varying loads (5, 7, and 10 N) at a sliding speed of 10 cm/s. It is evident that the wear depth and width are larger in PLA than in the PLA-HA composite. These wear profiles reflect the wear resistance of PLA-HA and reinforce the findings from the wear tests and 3D optical profilometer images.

### 3.2. Biodegradation of the Auxetic PLA-HA Scaffold

#### 3.2.1. Mass Loss and Swelling

**Figure 7:**
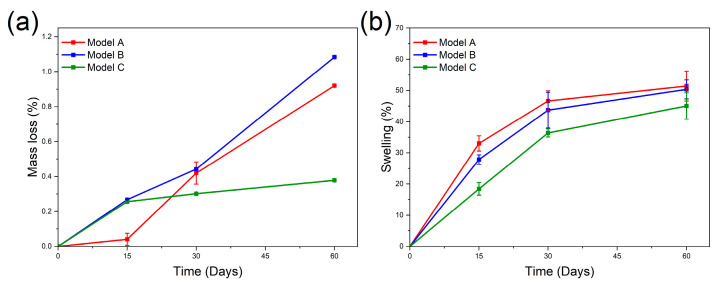
(**a**) Mass loss vs. Time and (**b**) Swelling vs. Time for 3D printed auxetic scaffolds (models A and B) and dense sample (model C) of PLA-HA composites after biodegradation tests in PBS solution.

Biodegradation of the PLA-HA composites was evaluated by soaking samples with the two different geometries (models A and B) and a dense sample (model C) in PBS to evaluate the changes in their properties. Understanding the biodegradation process of a biomaterial is crucial to determine the design and manufacturing parameters for the implants. Figure 7 shows the mass loss and swelling results of PLA-HA composites, all conducted in PBS solution at 37 °C. These results offer a comprehensive view of how each geometry (A, B, and C) responds to the simulated biological environment in terms of swelling and degradation.

Figure 7a depicts the mass loss of various auxetic scaffold geometries (models A and B) over 8 weeks (around 60 days) during biodegradation testing. The dense sample (model C) is included for comparison. All models show increased mass loss over time, indicating material degradation. During the first two weeks, the mass loss is low for all samples, continuing gradually over the first month. After this period, biodegradation accelerated, leading to higher mass loss, particularly in the auxetic scaffolds. Dense samples exhibit a slower degradation rate afterward. Model A, a more compact structure with less porosity, limits water penetration, thus reducing the degradation rate. Consequently, model A has a lower mass loss than model B, which has a more porous structure, exposing a larger internal surface area. This increased porosity facilitates faster biodegradation as degrading agents more easily penetrate the material, resulting in higher mass loss. This result demonstrates the significant role of porosity in the biodegradation rate of the auxetic structures. It is expected for in vivo test that presence of enzymes will boost the effect of porosity on degradation. Meanwhile, model C, being dense with low or no porosity, shows the greatest resistance to degradation due to less surface area contact, preventing effective penetration by degrading agents. This results in the lowest mass loss among the models. Similar behavior has been reported by other authors [63].

Figure 7b illustrates the swelling behavior of different scaffold geometries and the dense sample. All samples show rapid swelling rates up to 30 days, then continue to swell more slowly, reaching final values between 45% and 51%. The auxetic geometries have higher swelling ratios than the dense sample, with model A exhibiting the highest swelling. The greater specific surface area in auxetic structures promotes increased water uptake.

#### 3.2.2. Scanning Electron Microscopy (SEM)

**Figure 8:**
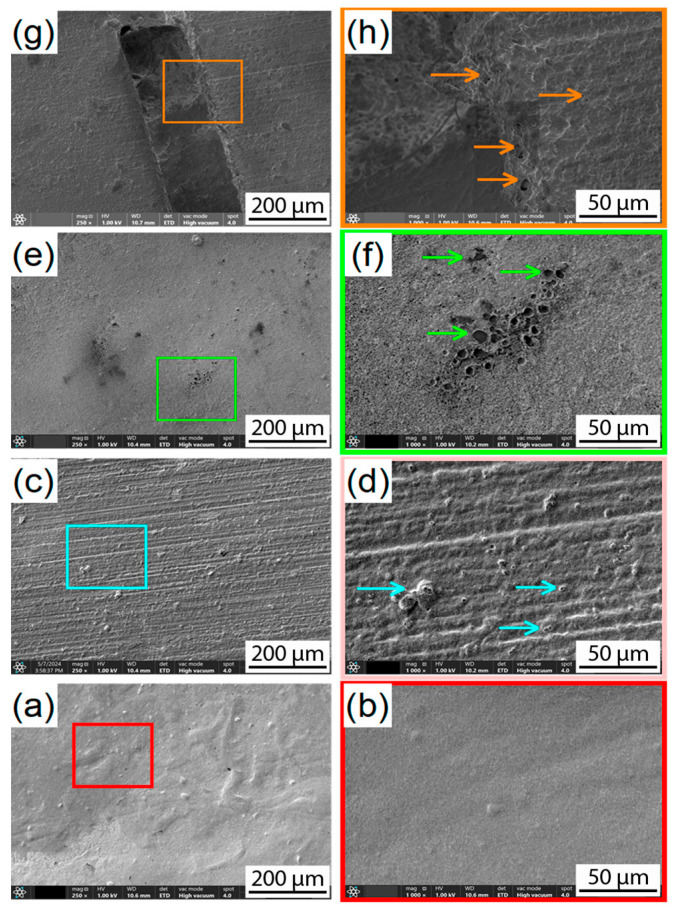
SEM micrographs at two different magnifications of PLA-HA composite after biodegradation test at various immersion times. At 0 days (**a**,**b**) is used as reference; after 15 days (**c**,**d**), reveals the particles (blue arrows); after 30 days (**e**,**f**), shows a higher degradation and porosity (green arrows); and after 60 days (**g**,**h**) massive degradation is observed (orange arrows).

The Scanning Electron Microscopy (SEM) examination was conducted solely on the PLA-HA composite specimen that experienced the biodegradation process. Figure 8 demonstrates the evolution of the surface morphology of the composite throughout the degradation period. The images designated “a” and “b” represent the specimen prior to the biodegradation process, serving as a reference point for assessing the structural integrity and surface features before degradation. Figure 8c,d depict the surface morphology after 15 days of immersion, where initial signs of deterioration, such as microcracks or surface roughness may be observed, revealing the HA particles (blue arrows) and reflecting the early effects of the biological environment on the material. Figure 8e,f illustrates the state of the composite after one month, exhibiting more evident morphological alterations including increased porosity and surface degradation (green arrows), indicative of the onset of breakdown under biological conditions. The final images, “g” and “h”, display the composite after two months of immersion, revealing substantial surface degradation, material loss, and heightened porosity (orange arrows), signifying ongoing biodegradation.

#### 3.2.3. XRD Study Following In Vitro Tests

**Figure 9:**
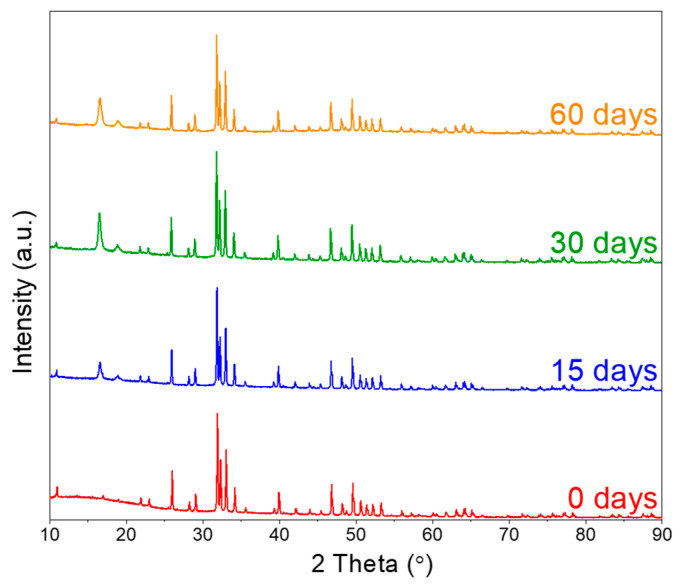
XRD patterns of the PLA-HA composite after biodegradation test at different immersion time.

Figure 9 displays the XRD patterns of 3D printed dense PLA-HA composites as a function of immersion duration following the biodegradation test conducted in PBS solution. Compared to the PLA-HA composite prior to immersion (0 days), a broad attenuation is observed within the 10–25° range, linked to the semicrystalline nature of PLA as discussed in Section 3.1, diminishes with increased immersion time. Moreover, a new peak emerges at 16.57°, which becomes more prominent over time. These observations are ascribed to the depolymerization of PLA into lactide through an alcoholysis reaction facilitated by hydroxyl groups originating from HA particles. Hu et al. [69] reported that PLA-HA composites subjected to cold sintering at various temperatures exhibit multiple crystalline regions of PLA resulting from the depolymerization of the polymeric chain under conditions of temperature, pressure, and the presence of HA. Additionally, the principal peaks of HA are noted to shift toward lower 2θ values, potentially indicating the formation of a new compound on the surface of the PLA-HA composite, as previously documented in the literature [70]. The slight shift observed in the XRD peak positions over immersion time can be further attributed to the hydrolytic degradation of the PLA matrix. During immersion in PBS, hydrolysis preferentially attacks the amorphous regions of PLA through ester bond scission, progressively enriching the relative crystalline fraction over time. The breakage of PLA chains into smaller segments due to hydrothermal chain scission increases their molecular mobility, allowing them to rearrange into crystalline domains more easily, resulting in an increase in the degree of crystallinity as a function of immersion time [71]. The selective degradation of the amorphous phase leads to structural rearrangements within the polymer matrix, which modify the diffraction profile and may induce subtle shifts in peak positions [72].

**Figure 10:**
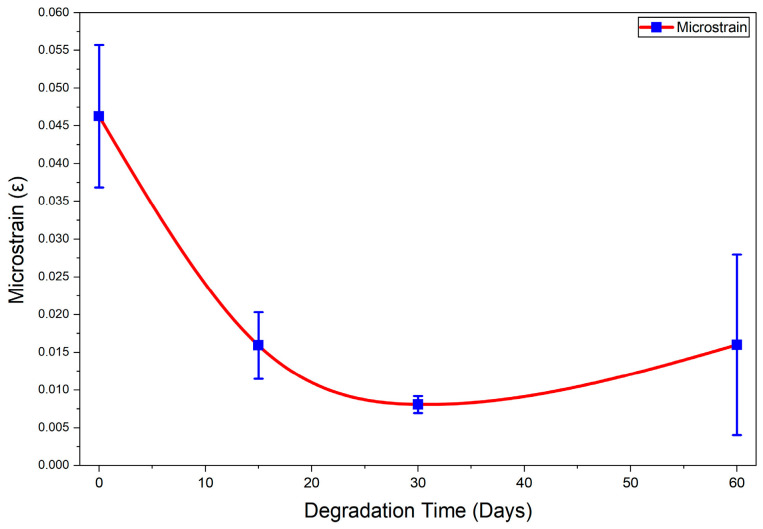
Microstrain evolution of PLA-Hydroxyapatite composite before degradation and after 15, 30, and 60 days of PBS immersion.

Figure 10 illustrates the evolution of microstrain in the PLA-Hydroxyapatite (PLA-HA) composite during hydrolytic degradation in PBS, as determined by the Williamson–Hall analysis [48]. The microstrain decreased significantly from 0.046 ± 0.009 at 0 days to 0.008 ± 0.010 after 30 days of degradation, corresponding to an overall reduction of approximately 85%. This pronounced decrease can be attributed to the progressive relaxation of residual internal stresses initially present within the composite. The incorporation of hydroxyapatite into the PLA matrix disrupts polymer chain packing and increases structural disorder, thereby generating residual microstrain in the as-prepared material [59]. Upon immersion in PBS, hydrolytic degradation predominantly affects the amorphous regions of the PLA matrix, which are more accessible to water molecules and more susceptible to chain scission than crystalline domains [73]. The preferential degradation of these defect-rich and highly strained regions progressively eliminates the primary sources of lattice distortion, leading to the observed reduction in microstrain. This interpretation is further supported by the preservation of more ordered crystalline domains, while disordered regions are selectively degraded [74].

At longer degradation times, a slight but noticeable increase in microstrain was observed after 60 days, rising from 0.008 to 0.016. This increase can be explained by the combined effect of two concurrent mechanisms occurring at advanced stages of degradation. First, hydroxyapatite particles may act as nucleating agents, promoting secondary recrystallization of low-molecular-weight PLA fragments generated during hydrolysis, which leads to the formation of imperfect crystalline structures and introduces new lattice distortions. Second, prolonged immersion induces heterogeneous bulk degradation, resulting in localized swelling, microcrack formation, and interfacial deterioration between PLA and hydroxyapatite. These phenomena generate new internal stress fields within the composite matrix [75,76]. The combined effect of these mechanisms accounts for the increase in microstrain observed at 60 days, indicating a transition from a stress relaxation-dominated regime to a microstructural damage-dominated regime at later stages of hydrolytic degradation.

#### 3.2.4. Hardness Following In Vitro Test

**Figure 11:**
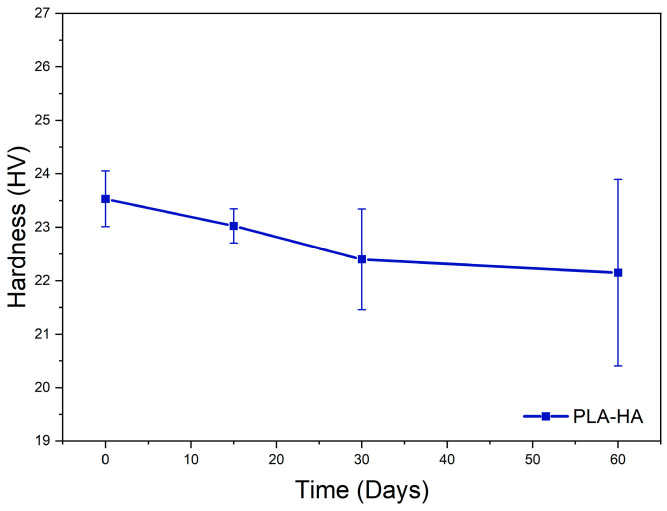
Hardness results of the 3D printing sample model C as a function of degradation time.

The results regarding the hardness of the 3D printed dense PLA-HA composite, following in vitro testing in PBS solution, are presented in Figure 11. At the initial stage, on day 0, model C demonstrates higher hardness values in comparison to the 3D printed neat PLA produced under identical conditions (19 HV). This enhancement in hardness is attributed to the presence of hard ceramic HA particles, which exhibit strong interfacial adhesion to the polymer matrix. Furthermore, the obtained results (23.53 ± 0.5 HV) align with those reported in prior research [77]. As degradation progresses, the composite’s resistance to deformation under applied load gradually diminishes until day 30, signifying the onset of the degradation process that affects the internal cohesion between HA and PLA. Subsequent to day 30, the hardness continues to decline at a reduced rate until day 60. The fluctuation in hardness observed after day 30 may denote premature degradation of the PLA matrix during the initial short period, followed by stabilization at a consistent rate thereafter. Furthermore, the relatively high standard deviation values for hardness are noted for immersion durations exceeding 30 days, implying a more heterogeneous structure post-degradation, characterized by increased porosity, diminished matrix integrity, and detachment of ceramic particles.

#### 3.2.5. Compression Test After In Vitro

**Figure 12:**
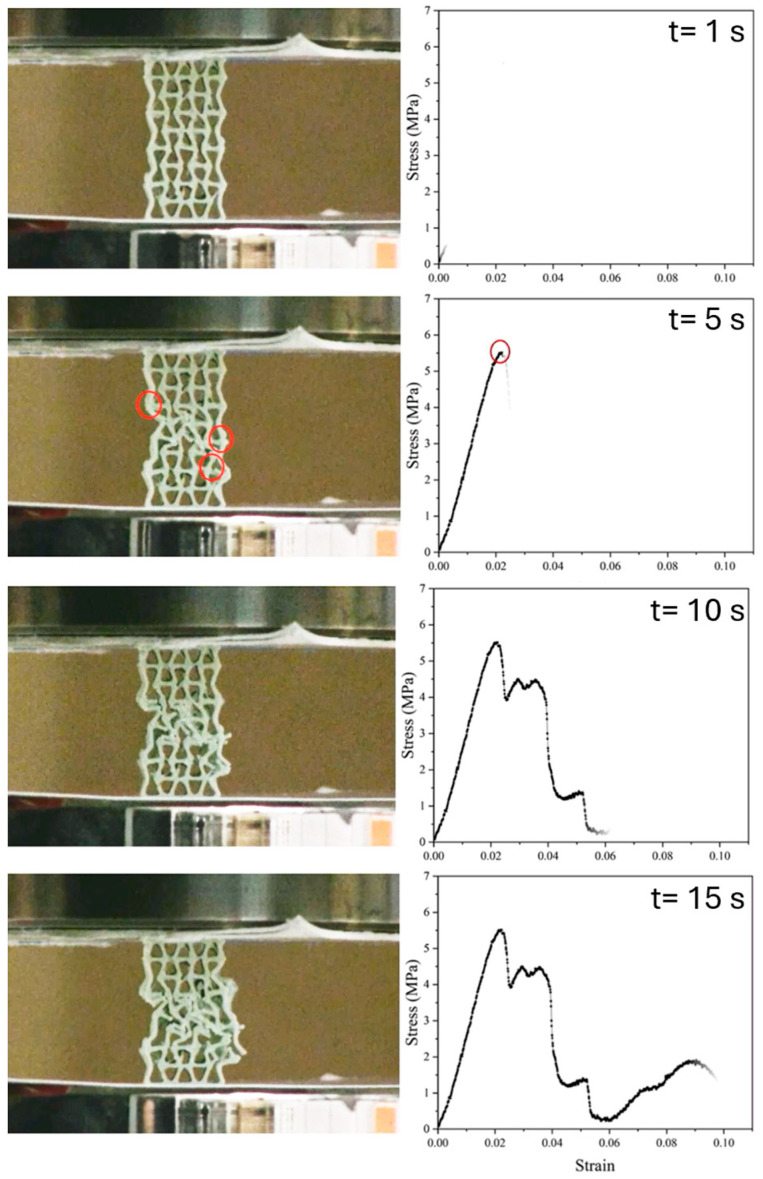
Video recording of the compression test on auxetic sample of model B without in vitro degradation (t = 0) and its synchronized stress–strain curve recorded during test. Complete video of the compression can be accessed in https://zenodo.org/records/19800236 (accessed on 26 April 2026) [78].

The results of the compression tests for two distinct geometries of auxetic scaffolds fabricated from the PLA-HA composite are presented. An illustrative example of the comprehensive compression test procedure can be observed in Figure 12, which displays the screenshots taken from the video of the compressed sample and synchronized with the stress–strain curve for one model B, without in vitro degradation (t = 0) [78]. It can be observed in the stress–strain curve that initially the sample exhibits elastic deformation with a gradual increase in stress, culminating in a peak at approximately 5.5 MPa after 5 s, which represents the ultimate compressive strength of the structure. At this juncture, the structure experiences a sudden decline in stress, signifying the onset of catastrophic failure. As highlighted within the red-circled region on both the stress–strain curve and the corresponding frame, the auxetic structure undergoes a localized collapse. The deformation pattern indicates buckling and subsequent fracturing of the internal struts within the lattice.

Despite the predominantly polymeric nature of the composite, the addition of hydroxyapatite (HA) enhances the stiffness of the structure, which likely contributed to stress localization and brittle fracture under compressive load. The auxetic geometry, while initially improving mechanical compliance and energy absorption, also resulted in stress concentration at specific nodes once large deformations were attained. Post-peak stress fluctuations indicate progressive failure mechanisms, such as layer-wise collapse or sequential buckling, within the architected structure. These findings highlight the importance of structural geometry and material composition in determining the failure behavior of printed auxetic materials.

The compression data for samples fabricated with the two models across biodegradation periods of 0, 15, 30, and 60 days are presented as stress versus strain curves, along with mean stress values, illustrating overall trends in the mechanical properties and auxetic behavior of these composites as biodegradation advances.

**Figure 13:**
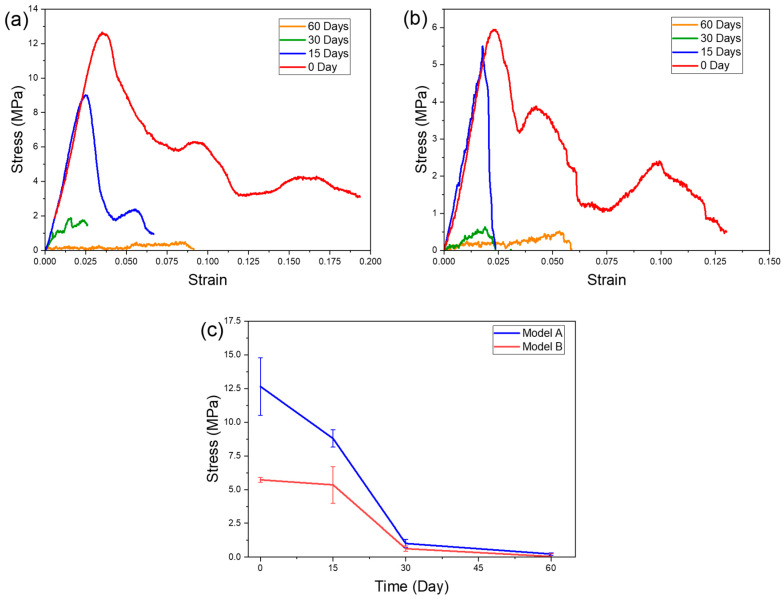
Evolution of mechanical properties as function of degradation time. (**a**) Model A and (**b**) model B and (**c**) mean value of the compression strength.

As observed from the stress–strain curves (Figure 13a,b) of the initial PLA-HA composite, a general trend of linear elastic behavior followed by oscillatory stages is evident for both auxetic geometries. In the elastic region, the stress increases proportionally with strain up to the maximum stress, with the walls undergoing slight bending at the onset of deformation. Subsequently, deformation is dominated by the bending and rotation of the cell walls within the auxetic structure. The crushing occurs layer by layer, resulting in oscillatory behavior and a non-uniform stress distribution. Furthermore, samples of model A, which possess lower porosity, demonstrate higher stress values and achieve larger strains prior to fracture.

Following 15 days of biodegradation, both geometries exhibit comparable elastic modulus values, as seen in the elastic segment of the curves. However, a slight decrease in maximum stress is observed, indicating a partial loss of structural integrity, likely attributable to initial superficial material degradation. At this stage, the PLA-HA composite largely retains its mechanical strength; however, degradation of the PLA phase may commence, impacting the overall structural integrity. Regarding auxetic behavior, the stress–strain curves display oscillatory behavior for model A under compression, implying the auxetic structure remains functional at this early biodegradation stage. Consequently, the samples maintain their fundamental mechanical properties and retain the ability to deform in an auxetic manner under load, with the PLA matrix continuing to provide the necessary flexibility for lateral deformation. Conversely, model B exhibits a direct decline in stress following the maximum, indicating embrittlement of the PLA-HA composite with geometry B.

At 30 days, a more significant alteration in the mechanical properties becomes evident from the compression curves. The maximum stress of the samples is substantially reduced compared to initial values, signifying a degradation of the scaffold’s mechanical integrity. The slope of the deformation curves becomes gentler, suggesting increased ductility and reduced resistance under compression. This decline can be ascribed to the progressive degradation of chemical bonds within the PLA-HA composite, especially affecting the PLA polymer’s rigidity. Auxetic behavior is also impacted, though the samples still exhibit some lateral deformability. Nonetheless, this deformation is less pronounced than at 15 days, implying partial degradation of the internal structures responsible for lateral expansion. This indicates a diminishing auxetic behavior concomitant with degradation of the PLA-HA composite, with associated loss of pore and microstructure integrity vital for auxetic deformation. The decline in mechanical properties observed in scaffolds with auxetic geometry aligns with those of dense samples.

The mechanical properties of the auxetic PLA-HA scaffolds, as depicted in Figure 13c, demonstrate a similar trend of progressive strength decline with increased immersion time. After 15 days, the properties remain relatively stable for model B with high porosity, whereas significant degradation is evident for model A with low porosity. As biodegradation extends to 30 and 60 days, the samples progressively lose stiffness. These findings highlight the delicate balance between material degradation, which facilitates biological integration, and the reduction in mechanical performance, an essential consideration for biomedical applications requiring scaffolds to support loads over a designated period before degradation.

**Figure 14:**
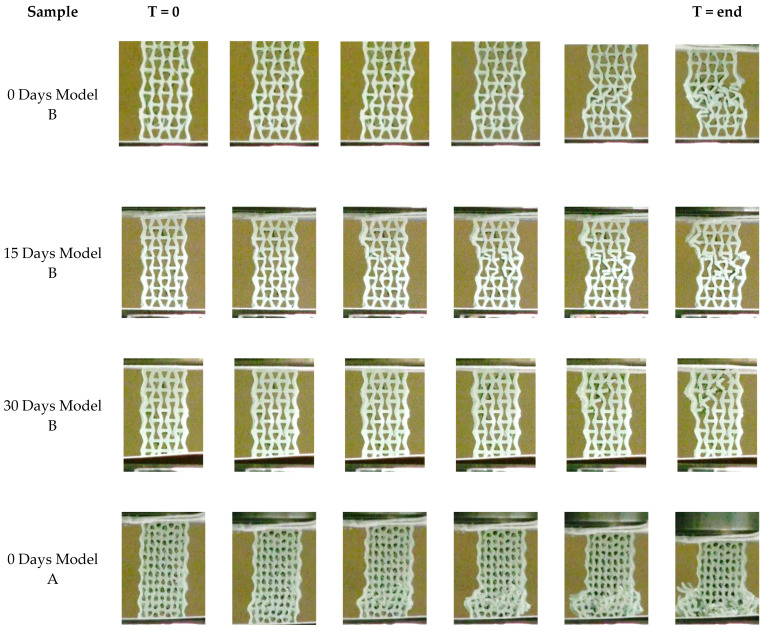

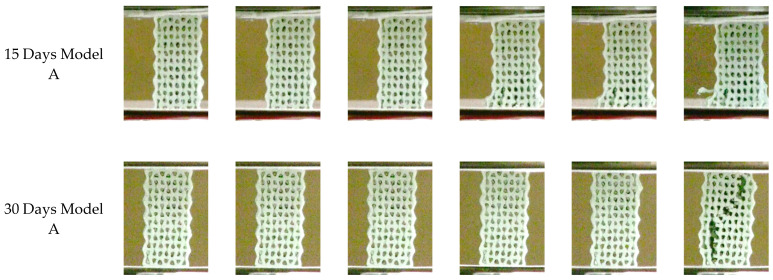
Evolution of fracture in the auxetic structures before the degradation experiments. Pictures are taken from the video at different times of the compression test. (Accelerated videos of the whole test are in the Supplementary Materials).

The evolution of the fracture during the compression test is depicted in Figure 13, both prior to degradation and after 15 and 30 days of degradation. The images were captured at various intervals during the compression test. The results suggest that, before degradation, the fracture manifested layer by layer. For geometry A, deformation initiates in the lower cells, whereas for geometry B, it begins in the central cells, as illustrated in Figure 14. This behavior is attributable to the relatively weak lateral stiffness of model B compared to model A; consequently, model B can deform centrally owing to the limited common support between the cells. With regard to the stress–strain curves, they exhibit a sinusoidal pattern, increasing, then decreasing, rising again, and gradually declining until ultimate failure. After 15 days of degradation, geometry A maintains the same deformation pattern, as shown in the images indicating layer-by-layer fracture. Conversely, the fracture pattern for geometry B changes to a vertical or slightly inclined shear mode, aligned with the orientation of the auxetic cell walls. This transition accounts for the abrupt failure observed immediately after reaching maximum strength, as depicted in Figure 14. The images documented after four weeks of degradation elucidate the deterioration of mechanical properties illustrated in the curve of Figure 10, where both models display shear failure.

## 4. Conclusions

This study evaluated 3D printed auxetic PLA-HA (40 wt%) composite scaffolds fabricated via Fused Filament Fabrication. The incorporation of HA into the PLA matrix significantly improved the material’s hydrophilicity and enhanced its mechanical and tribological properties, specifically increasing hardness and elastic modulus. Furthermore, tribological assessments confirmed enhanced wear resistance for the composite under dry conditions.

In vitro degradation analysis demonstrated that the geometric design of the auxetic structures heavily influences degradation kinetics. The higher porosity structure (model B) exhibited accelerated degradation compared to the less porous structure (model A), confirming that architectural design can be used to dictate material resorption rates. Over time, progressive degradation led to observable changes in surface morphology and a gradual decline in mechanical resistance.

These findings show that combining HA reinforcement with tailored re-entrant auxetic geometries allows for the customization of mechanical and degradation profiles.

## Figures and Tables

**Table 1:** The geometric parameters for auxetic re-entrant cells A and B.

Designation	Length, L (mm)	Width, W (mm)	Angle θ (°)	Wall Thickness, T (mm)
A	3.00	1.78	70	0.4
B	4.00	2.31	70	0.4

## Data Availability

The original contributions presented in this study are included in the article/Supplementary Materials. Further inquiries can be directed to the corresponding authors.

## Supplementary Materials

The following supporting information can be downloaded at: https://www.mdpi.com/article/10.3390/ma19101972/s1, Video S1: Speedy Video (×500) of the compression test for model A after 0 days of degradation 00366; Video S2: Speedy Video (×500) of the compression test for model A after 15 days of degradation 00373; Video S3: Speedy Video (×500) of the compression test for model A after 4 weeks of degradation 00381; Video S4: Speedy Video (×500) of the compression test for model A after 4 weeks of degradation 00389.; Video S5: Speedy Video (×500) of the compression test for model B after 0 days of degradation 00366; Video S6: Speedy Video (×500) of the compression test for the model B after 15 days of degradation 00373; Video S7: Speedy Video (×500) of the compression test for model B after 4 weeks of degradation 00381; Video S8: Speedy Video (×500) of the compression test for model B after 4 weeks of degradation 00389.
