# Supplementary material for: Design and Characterization of 3D Printed Auxetic PLA-HA Composite Scaffolds for Biomedical Application
Source: Materials (Basel). 2026 May 10;19(10):1972. doi: 10.3390/ma19101972 (PMC13208656; doi:10.3390/ma19101972)
Supplement: Supplementary file 1 [file materials-19-01972-s001.zip › materials-4241706-supplementary.pdf]

Design and Characterization of 3D Printed Auxetic PLA-HA Composite Scaffolds for Biomedical Application

Mohammed Amine Benziada [1,*], Antonio Javier Sanchez-Herencia [2,*], Isamil Daoud [1], Hossein Besharatloo [3,4], Begoña Ferrari [2], Djamel Miroud [1], and Ana Ferrandez-Montero [2]

[1] Laboratory of Materials Sciences and Engineering (LSGM), University of Sciences and Technology Houari Boumediene, Bab Ezzouar, Algiers 16111, Algeria; ismail.daoud@usthb.edu.dz (I.D.); dmiroudlsgm18@gmail.com (D.M.)
[2] Instituto de Ceramica y Vidrio (ICV-CSIC), Calle Kelsen 5, 28049 Madrid, Spain; bferrari@icv.csic.es (B.F.); aferrandez@icv.csic.es (A.F.-M.)
[3] Centre d'Integritat Estructural, Fiabilitat i Micromecànica dels Materials (CIEFMA-UPC), Department of Materials Science and Engineering, Universitat Politècnica de Catalunya—BarcelonaTech, Campus Diagonal Besòs-EEBE, 08019 Barcelona, Spain; hossein.besharatloo@upc.edu (H.B.)
[4] Barcelona Research Center in Multiscale Science and Engineering, Universitat Politècnica de Catalunya—BarcelonaTech, Campus Diagonal Besòs, 08019 Barcelona, Spain
* Correspondence: mbenziada@usthb.edu.dz (M.A.B.); ajsanchez@icv.csic.es (A.J.S.-H.)

Links to Supplementary Material

Supplementary Video S1. Speedy Video (x500) of the compression test for the Model A after 0 days of degradation 00366. https://doi.org/10.5281/zenodo.19133108

Supplementary Video S2. Speedy Video (x500) of the compression test for the Model A after 15 days of degradation 00373. https://doi.org/10.5281/zenodo.19134133

Supplementary Video S3. Speedy Video (x500) of the compression test for the Model A after 4 weeks of degradation 00381. https://doi.org/10.5281/zenodo.19134270

Supplementary Video S4. Speedy Video (x500) of the compression test for the Model A after 8 weeks of degradation 00389. https://doi.org/10.5281/zenodo.19134344

Supplementary Video S5. Speedy Video (x500) of the compression test for the Model B after 0 days of degradation 00366. https://doi.org/10.5281/zenodo.19134442

Supplementary Video S6. Speedy Video (x500) of the compression test for the Model B after 15 days of degradation 00373. https://doi.org/10.5281/zenodo.19134535

Supplementary Video S7. Speedy Video (x500) of the compression test for the Model B after 4 weeks of degradation 00381. https://doi.org/10.5281/zenodo.19134613

Supplementary Video S8. Speedy Video (x500) of the compression test for the Model B after 4 weeks of degradation 00389. https://doi.org/10.5281/zenodo.19134683
